# Spatial-Temporal Variations and Trade-Offs of Ecosystem Services in Anhui Province, China

**DOI:** 10.3390/ijerph20010855

**Published:** 2023-01-03

**Authors:** Qiangqiang Yang, Pian Zhang, Xiaocong Qiu, Guanglai Xu, Jianyu Chi

**Affiliations:** 1 School of Civil and Hydraulic Engineering, Ningxia University, Yinchuan 750021, China; 2 School of Management Engineering, Wanjiang University of Technology, Maanshan 243000, China; 3 School of Life Sciences, Ningxia University, Yinchuan 750021, China; 4 School of Geography and Tourism, Anhui Normal University, Wuhu 241003, China; 5 Collaborative Innovation Center of Recovery and Reconstruction of Degraded Ecosystem in Wanjiang Basin Co-founded by Anhui Province and Ministry of Education, Anhui Normal University, Wuhu 241003, China

**Keywords:** ecosystem services, trade-off/synergy, InVEST model, PLUS model, Anhui Province

## Abstract

Research on the spatiotemporal evolution and trade-offs of ecosystem services (ESs) is important for optimizing the ecological security barrier system and promoting coordinated socio-economic development. Natural factors, e.g., climate change, and human factors, e.g., unreasonable land use, have impacted and damaged ecosystem structure and function, leading to challenges with ES trade-offs and the spatial identification of priority protected areas. Here, the spatiotemporal evolution characteristics of five ESs (water yield, nitrogen export, soil retention, carbon storage, and habitat quality) in Anhui Province, China, from 2000–2020 were analyzed based on the Integrated Valuation of Ecosystem Services and Trade-offs (InVEST) model. The trade-offs and spatial patterns among different ESs were explored using Pearson correlation and hotspot analyses; the dynamics of natural growth, cultivated land protection, and ecological protection scenarios for ESs in 2030 were simulated and analyzed by coupling InVEST with the patch-generating land use simulation (PLUS) model. The results reveal the following. (1) From 2000–2020, increases in water yield and soil retention occurred, with concurrent declines in the other services; the total nitrogen high-value area was mainly concentrated in the plain, with the other services’ high-value areas mainly concentrated in the Dabieshan and Southern Anhui Mountains, with each ES showing similar spatial distributions across years. (2) The ESs were mainly synergistic, with trade-offs mainly between nitrogen export and other services. (3) Hotspot overlap between water yield and the other ESs was relatively low; no more than 6.53% of ecosystems per unit area provided five ESs simultaneously. (4) Other than water yield, the ecological protection scenario was more conducive to improving ecosystem functions. This study’s results indicate inadequate synergy among ESs in Anhui Province; competition among land types must be further balanced in the future. This study provides a basic reference for implementing ecological projects and constructing ecological security patterns.

## 1. Introduction

Ecosystem services (ESs) are defined as various benefits that humans obtain either directly (e.g., water conservation) or indirectly (e.g., waste disposal) through the structure, process and function of an ecosystem [1,2,3]. Research on ESs is an effective way to address sustainability challenges and maintain human wellbeing [4]. With the rapid advancement of the social economy and living standards, increasing carbon emissions [5], water pollution [6], soil erosion [7], and biodiversity losses [8] have resulted in more than 60% of ecosystems being degraded or unsustainably used [9], which directly affects the structure and function of ecosystems and ecological security [10]. Consequently, the coupling of ESs with analyses of changes in land use and the associated driving factors has become a topic of major interest and a core focus of ecosystem research [11].

Land is the spatial carrier of human survival and major socioeconomic activities [12,13,14], and human activities have led to varying degrees of change on more than 70% of the global land surface [15], intensifying competition among different land use types and threatening ecological security [16,17]. However, land use and land cover changes (LUCCs) are closely related to ESs. Irrational LUCCs alter landscape patterns and ecological processes, leading to ES degradation, and ESs counteract land use efficiency, such that LUCCs and ESs interact and influence each other [18]. For example, due in part to the forest restoration policy, the carbon storage capacity of the Yangtze River Delta region initially increased due to the conversion of grassland to forest land; with economic development and urban expansion, the proportion of construction land increased and the carbon sequestration capacity of the region decreased; eventually, the expansion of construction land was restricted in the context of ecological civilization construction and development [19]. Scholars and decision-makers have developed strategies for land use protection from different perspectives to promote the sustainable development of regional ESs [20]. However, ESs have uneven spatial distributions, and the relationships between ESs are complex and intertwined, with various synergies and trade-offs [21]. Scholars have attempted to weaken the impact of trade-offs between ESs on coordinated regional development by seeking potential high-value areas. Bai et al. [22] analyzed the spatial distribution characteristics of high-value areas for biodiversity and other ESs at the watershed scale. Benis et al. [23] explored the overlap between different target levels of ES hotspots and biodiversity priority areas in South African grasslands and analyzed the possibility of combining the two types of regions. Further in-depth research must be performed to determine how to construct multiobjective ES priority areas to reconcile trade-offs between services [24].

Currently, the main ecological models for assessing ESs include SWAT [25], ARIES [26], RUSLE [7], SolVES [27], and InVEST [8]. The Integrated Valuation of Ecosystem Services and Trade-offs (InVEST) model covers a variety of ES processes and offers the advantages of flexible parameters, high stability, and a strong visualization ability over other models [28,29]. This model provides powerful technical support for ecosystem management and is widely used in the assessment of ESs [30]. For instance, Wang et al. [30] applied the habitat quality module of InVEST to different geomorphic types in Xinjiang and found that mountainous areas outperform plains, and Li et al. [31] used the InVEST model to analyze the spatial and temporal variability of ESs in the Hexi Region and identified the trade-offs between different services based on correlation analysis. In addition, scholars have dynamically simulated multi-scenario land use patterns through predictive models to optimally allocate land resources. In particular, the Patch-generating Land Use Simulation (PLUS) model proposed by Liang et al. [32] in 2020 can predict land use changes and analyze its drivers by integrating the land extension analysis strategy (LEAS) and the CA model based on multiple types of random seeds (CARS) and extracting various types of land use extensions between two land use change images, has higher simulation accuracy than existing software (e.g., CLUE-S [33], FLUS [34], and ANN-CA [35]), and has been effectively applied for land use simulations and predictions. Gao et al. [36] considered Nanjing as an example to explore the ecological risks of land use under different development scenarios using the PLUS model and ecological risk indicators and provided technical support for ecological risk management. Wang et al. [37] used the PLUS and InVEST models to simulate changes in future land use patterns and the regional carbon storage capacity and found that scenario establishment had a significant influence on the accuracy and reliability of the simulation results. The coupled PLUS-InVEST model can be applied to simulate changes in land use and ES assessment to provide useful guidance for improving regional ESs [38].

Anhui Province is located in the hinterland of the Yangtze River Delta, with diverse landform types, abundant hydrothermal resources, and an outstanding ecological location. As urbanization and high-quality integrated development of the Yangtze River Delta have resulted in a sharp increase in environmental pressure, elucidating changes in ES functions is very useful for the green development of the region [38]. In this study the InVEST (v. 3.11.0) and PLUS (v. 1.3.6) models are applied to Anhui Province as a research subject to (1) elucidate the spatiotemporal dynamics of ESs; (2) clarify the trade-offs and synergies among ESs; (3) identify priority protected areas; and (4) predict future land use patterns and explore development trends of ESs.

## 2. Materials and Methods

### 2.1. Study Area

**Figure 1:**
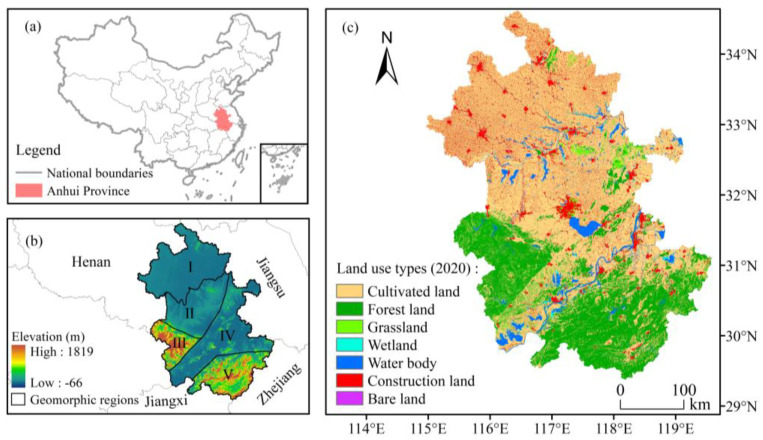
(**a**) Location of Anhui Province, China; (**b**) elevation and geomorphic zoning: I: Huaibei Plain; II: Jianghuai Plain; III: Dabieshan Mountain in West Anhui; IV: a hilly plain in the Yangtze River region; V: mountains in South Anhui; and (**c**) land use of the study area in 2020.

Anhui Province is located in the hinterland of East China (114°54′ E–119°37′ E, 29°41′ N–34°38′ N). It has jurisdiction over 16 prefecture-level cities and, as of the end of 2020, had a resident population of 61.05 million, an urbanization rate of 58.33%, and a gross domestic product of 386.81 billion yuan. The study area has a total area of 140,100 km^2^, and the main land use is cultivated land, followed by forest land and construction land. The area has an average annual precipitation of 800–1800 mm and an average annual temperature of 14–17 °C. The overall terrain in the study area is high in the southwest and low in the northeast and divided from north to south into five geomorphic regions [39] (Figure 1) spanning the Huaihe River, Yangtze River, and Xin’an River Basin. Among them, the north Huaihe River has a warm temperate semihumid monsoon climate and is dominated by a wheat–maize (soybean) rotation, and the south Huaihe River has a subtropical humid monsoon climate and is dominated by a wheat (canola)–rice rotation [40]. As one of the main entities responsible for promoting the integrated development of the Yangtze River Delta, Anhui Province is a comprehensive hub connecting cities in the east and west, and the quality of its ecological environment is important for protecting the sustainable development lifeline of the Yangtze River Delta and exploring an eco-friendly and high-quality development model [41].

### 2.2. Data Sources

Land use data with a resolution of 30 m for 2000, 2010, and 2020 were acquired from GlobeLand30 (http://globeland30.org, accessed on 6 May 2022) produced by the National Geomatics Center of China. This dataset includes a total of 10 first-level types, and the overall classification accuracy is greater than 83% (2010 and 2020). This global land cover dataset has the highest resolution and best classification accuracy available to the public [42,43]. According to the actual situation of the study area, the land use composition was divided into seven categories: cultivated land, forest land, grassland, wetland, water body, construction land, and bare land. A digital elevation model (DEM) with a resolution of 30 m was obtained from ASTER GDEM3 grid data (https://www.earthdata.nasa.gov, accessed on 4 May 2022). Data with a resolution of 1 km (the monthly precipitation, temperature, and potential evapotranspiration) and grid data with a resolution of 30 m (the rainfall erosivity (R) [44] and soil erodibility (K) [45]) were acquired from the National Earth System Science Data Center (http://www.geodata.cn). Soil property data were obtained from the Harmonized World Soil Database v 1.2 (https://www.fao.org/soils-portal/en, accessed on 30 June 2022). The data were resampled by ArcGIS (ESRI, version 10.8) software to a consistent resolution of 30 m, and WGS_1984_UTM_Zon_50N was used consistently as the spatial reference system.

### 2.3. ES Quantification

**Figure 2:**
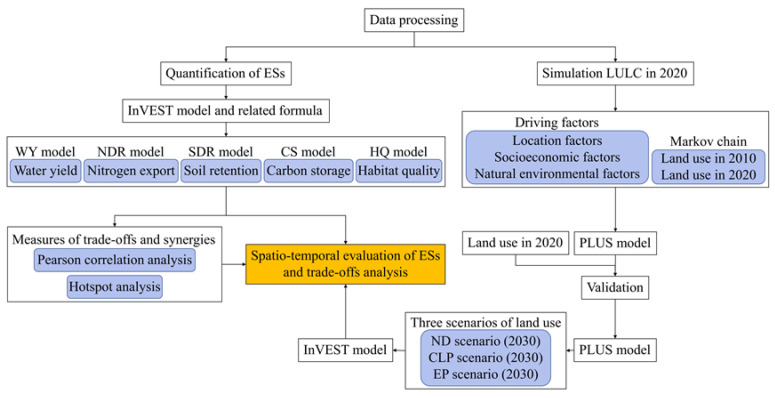
The research framework used in this study.

The analytical framework used in this study is shown in Figure 2.

#### 2.3.1. ES Selection

The Red Line of Ecological Protection in Anhui Province published in June 2018 points out several ecological problems in the province, such as the need to improve forest ecological functions, the irrational exploitation of river and lake wetlands, prominent soil erosion issues, and threats to biodiversity, which impact the stability of the ecosystem structure and the complexity of its functions, leading to prominent conflicts between resource utilization and resource protection and restoration. To address the existing problems, this study selected five ESs, namely, the water yield, nitrogen export, soil retention, carbon storage, and habitat quality, based on the InVEST model to investigate the characteristics of the spatiotemporal evolution and trade-offs of ESs in the study area.

#### 2.3.2. Water Yield

The Annual Water Yield model was used to assess and analyze the water yield in Anhui Province based on the Budyko curve, the water balance principle [46] (consisting of the annual precipitation minus the actual annual evapotranspiration [18,47]), and the salient parameters, such as the precipitation, reference evapotranspiration [48], root depth, Z parameter (7.8) [49,50], plant available water fraction [25] and land use types. The water yield depth of each land use type was extracted using Zonal Statistics as a table tool in ArcGIS. The main formula can be expressed as follows:

(1) $$Y\left(x\right)=\left[1-\frac{AET\left(x\right)}{P\left(x\right)}\right]\times P\left(x\right)$$

where *Y*(*x*) represents the annual water yield of pixel *x* (mm); *P*(*x*) indicates the annual precipitation of pixel *x* (mm); and *AET*(*x*) represents the annual actual evapotranspiration (mm).

#### 2.3.3. Nitrogen Export

The Nutrient Delivery Ratio model estimates the output of pollutants based on the mechanism by which vegetation and soil can convert or store nitrogen or phosphorus pollutants in runoff [51]. In this study, the total nitrogen output per unit area was used to characterize the capacity of water purification services. The lower the total nitrogen output per unit area is, the higher the water purification service capacity of an ecosystem is [52]. Nutrient loads, retention efficiencies, and maximum retention lengths were obtained from the literature [53,54]. The main formula can be expressed as follows:

(2) $$ALV_{x}=HSS_{x}\times pol_{x}$$

where *ALV_x_* represents the adjusted load value of a pixel unit *x*; *HSS_x_* represents the hydrological sensitivity score value of a pixel unit *x*; and *pol_x_* represents the pollutant export coefficient of a pixel unit *x*.

#### 2.3.4. Soil Retention

Soil conservation services reveal the ability of ecosystems to control erosion and retain sediment [55]. The Sediment Delivery Ratio model calculates soil retention based on the difference between potential and actual soil erosion [55,56]. Among the parameters required to run the model, the cover-management factor (*C*) and support practice factor (*P*) were obtained from the literature [54]. The formulas are given as follows:

(3) SR=Ap−Ar=R×K×L×S×(1−C×P)

where *SR* refers to the amount of soil conservation (t/hm^2^), *Ap* and *Ar* are the potential soil loss (t/hm^2^) and actual soil loss (t/hm^2^); *R* represents the rainfall erosivity factor (MJ·mm·h^−1^·hm^−2^·a^−1^); *K* denotes the soil erodibility factor (t·hm^2^·h·MJ^−1^·hm^−2^·mm^−1^); *LS* is the slope length-gradient factor; *C* and *P* are the cover-management factors and the support practice factor (unitless).

#### 2.3.5. Carbon Storage

The Carbon Storage and Sequestration model quantifies carbon storage by simplifying the carbon cycle process and combining land use types and carbon densities of different land classes [57,58]. In this study, the total carbon storage was calculated by summing the products of the areas of different land use types and the average carbon densities of the corresponding carbon pools, where the carbon densities were mainly obtained from previous studies [5,19,59,60]. The main calculation formula is as follows:

(4) $$C_{i}=(C_{i\_above}+C_{i\_below}+C_{i\_soil}+C_{i\_dead})\times S_{i}$$

where *C_i_* is the total carbon storage of land cover category *i* (t·hm^−2^·a^−1^); *C_i_above_*, *C_i_below_*, *C_i_soil_*, and *C_i_dead_* denote the above ground, below ground, soil and dead organic matter carbon density of land use category *i*, respectively (t/hm^2^); and *S_i_* represents the area of land use type *i* (hm^2^).

#### 2.3.6. Habitat Quality

Field studies can be used to quantitatively evaluate the quality and survival suitability of biological habitat more accurately than simulations but have limitations, such as operational difficulty, the inability to be used to generalize and compare evaluation results, and the ability to evaluate only a small range of specific targets [8]. The InVEST model has been widely used to estimate biodiversity as a reflection of the impacts of human disturbance on the ecological environment [61]: the higher the intensity of human activities is, the lower the habitat quality and biodiversity are [30]. There are five levels of habitat quality scores: low (0–0.2), relatively low (0.2–0.4), moderate (0.4–0.6), relatively high (0.6–0.8), and high (0.8–1.0) [62]. The main formula is as follows:

(5) $$Q_{xj}=H_{j}\left(1-\frac{D_{xj}^{z}}{D_{xj}^{z}+k^{z}}\right)$$

where *Q_xj_* is the habitat quality of grid cell *x* in land use type *j*; *H_j_* is the habitat suitability of land use type *j*; *D_xj_* is the total stress level on grid cell *x* in land use type *j*; *k* is the half-saturation constant, which is set to half of the highest grid pixel degradation value on the landscape; and *z* is a normalized constant, which is usually set to 2.5. The half-saturation constant (*k*) is half of the highest grid cell degradation value on the landscape. The value was set to 0.16 in this study. The biophysical data used in the InVEST model are shown in Table 1.

### 2.4. Trade-Off Analysis

Pearson’s correlation analysis was used to quantitatively analyze the degree of linear correlation between the ESs. A positive correlation coefficient indicates a synergistic (mutual gain) relationship between services, and a negative value indicates a trade-off relationship [63]. Specifically, the ArcGIS Create Random Points tool was used to create 1000 points and extract the ES value corresponding to each point, and the results were then analyzed.

### 2.5. Hotspot Analysis

A “hotspot” is an area that provides large contributions to a particular service [22]. Research on the identification of hotspots can indicate the strength of the service supply capacity in different regions, which is important for biodiversity conservation, the establishment of national parks, and precise spatial planning [64]. In this study, the high-value area of each ES corresponding to 30% of the size of the study area was defined as a hotspot.

### 2.6. Simulation of Changes in Land Use

**Figure 3:**
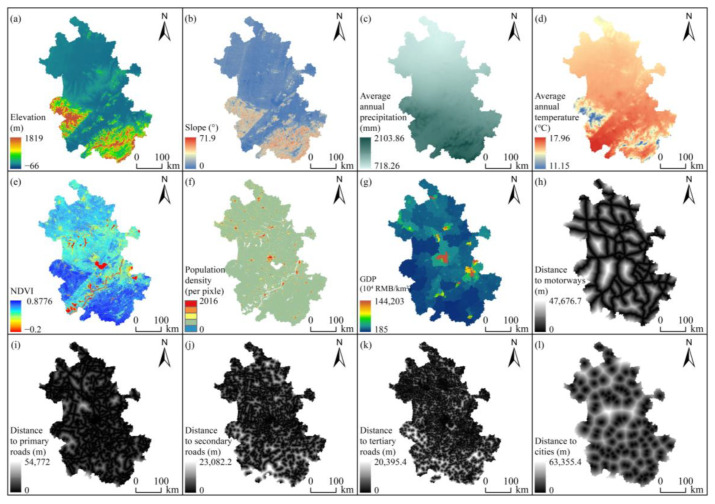
Driving factors for changes in land use.

The Markov-PLUS coupling model was used to determine the land use in 2020 based on land use data from 2010 to 2020, the driving factors (natural environmental factors: elevation, slope, average annual precipitation, average annual temperature [48], NDVI [65]; socioeconomic factors: population density (http://www.worldpop.org, accessed on 13 May 2022), GDP (http://www.resdc.cn, accessed on 14 September 2022); and location factors: distance to motorways, primary roads, secondary roads, tertiary roads, and cities (http://www.openstreetmap.org, accessed on 12 September 2022) (Figure 3) and the conversion constraint area (nature reserves). The accuracy and consistency of the simulation results were checked against the actual land use data in 2020. The validation results indicate that the overall accuracy and kappa coefficient were 0.890286 and 0.813871, respectively, which met the research needs [66]. Based on the actual characteristics of the study area, land use patterns for 2030 were simulated under the scenarios of natural development (ND, no restrictions), and cultivated land protection (CLP, where cultivated land and construction land cannot be converted to other land use types) and ecological protection (EP, where forest land, grassland, wetland and water bodies can be converted to each other but not to other land use types), and changes to future ESs under different scenarios were explored by coupling to the InVEST model. The neighborhood weights (calculated from the ratio of the expansion area of the land use type to the total land expansion [32]) and the transition matrix are shown in Table 2 and Table 3, respectively. 

## 3. Results

### 3.1. Changes in Land Use

**Figure 4:**
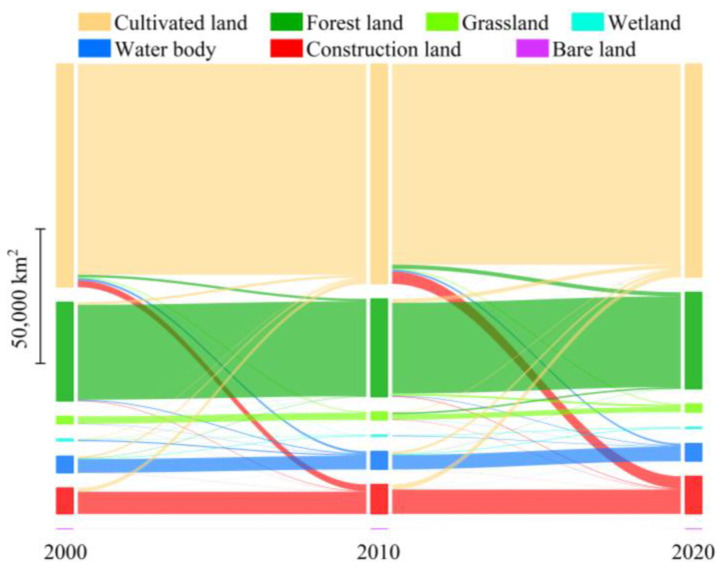
Sankey diagram of metrics for land use transfer in Anhui Province from 2000 to 2020.

The Sankey diagram (Figure 4) based on the land use transfer metric reveals the characteristics of the land use structure and the conversion relationship among the different land types in Anhui Province over the past 20 years. Overall, cultivated land was the main land use type in the study area and was primarily distributed in the Huaibei Plain, Jianghuai Plain, and Hilly Plain along the Yangtze River, followed by forest land and construction land, with forest land being mainly distributed in the Dabieshan and Southern Mountains. The three together accounted for more than 92.5% of the total area, and the area of bare land was the smallest, accounting for less than 0.04% of the total. In terms of the land use change trends from 2000 to 2020, construction land grew the most, with an increase of 4332.5 km^2^, and was mainly converted from cultivated land, followed by water bodies and grassland, with an increase of 364.1 km^2^ and 166.5 km^2^, respectively. In contrast, the areas of cultivated land and forest land continuously decreased, with decreases of 3715.6 km^2^ and 837.6 km^2^, respectively, and forest land was mainly converted to cultivated land. The other land types fluctuated with a small amplitude.

### 3.2. ES Distribution and Changes

**Figure 5:**
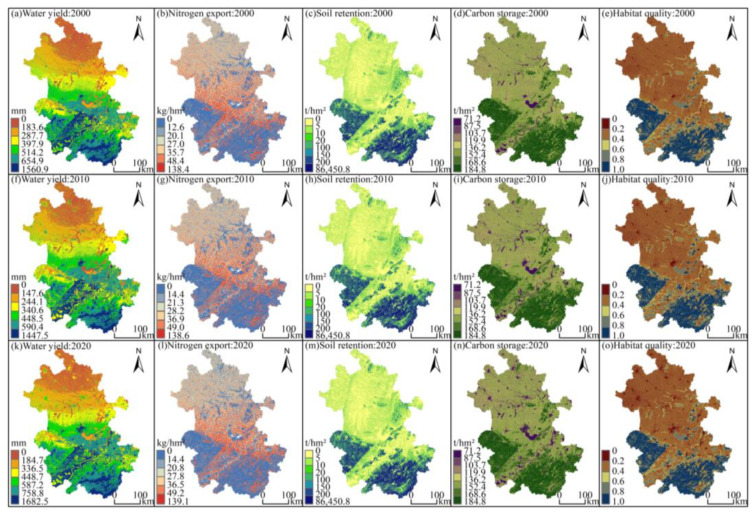
Spatiotemporal distribution of ESs in Anhui Province from 2000 to 2020.

From 2000 to 2020, strong spatiotemporal heterogeneity was observed among the ESs (Table 4 and Figure 5). In terms of spatial distribution, the water yield depth exhibited an overall distribution pattern of “being high in the south and low in the north, decreasing from south to north.” In addition, the average annual water yield depths of cultivated land, forest land, grassland, wetland, water body, construction land, and bare land were 400.2 mm, 444.8 mm, 528.0 mm, 244.9 mm, 152.2 mm, 608.7 mm and 523.2 mm, respectively, with those of construction land and water body being the highest and the lowest, respectively. From the time scale analysis, the total water yield increased but fluctuated, from 58.97 billion m^3^ in 2000 to 66.47 billion m^3^ in 2020, with an increase of 12.72%.

The average nitrogen outputs were 22.89 kg/hm^2^, 22.97 kg/hm^2^, and 22.79 kg/hm^2^, respectively, first increasing and then decreasing but decreasing overall, which was consistent with the change in the total nitrogen output, and the water purification service was improved. In particular, the high nitrogen loading area was relatively concentrated in the Jianghuai Plain and Hilly Plain along the Yangtze River and relatively sparsely concentrated in the Dabieshan and Southern Mountains area, mainly because its pollution load was small and the forest land and grassland had a high retention efficiency for nitrogen, which inhibited the diffusion of pollutants.

The sediment output decreased overall. The average soil retention intensity increased by 0.07 t/hm^2^, and the soil retention function increased by 1 million tons in 2020 compared to 2000. The soil retention per unit area of forest land was approximately 431.8 t/hm^2^, which was considerably higher than that of the other land types, followed by grassland (107.3 t/hm^2^), forming a spatial distribution pattern of high soil retention intensity in mountainous areas.

The spatial distribution pattern of carbon storage was roughly the same, with the high-value areas mainly concentrated in mountainous forest areas. The total quantity of carbon storage decreased over time, with a decrease of 33.84 million tons in 20 years, mainly due to the conversion of forest land and cultivated land with a high carbon density to construction land, which reduced the carbon storage capacity of the ecosystem.

In 2000, 2010, and 2020, the average habitat quality of the study area was 0.454, 0.453, and 0.440, respectively, and the sum of the “low”, “relatively low”, and “moderate” classes accounted for 72.16%, 71.82%, and 72.78% of the total study area, respectively, indicating that the overall habitat quality was moderate or lower and the level of biodiversity decreased due to increased disturbances from human activities in the region.

### 3.3. Trade-Offs and Synergies among ESs

#### 3.3.1. Correlation Analysis

**Figure 6:**
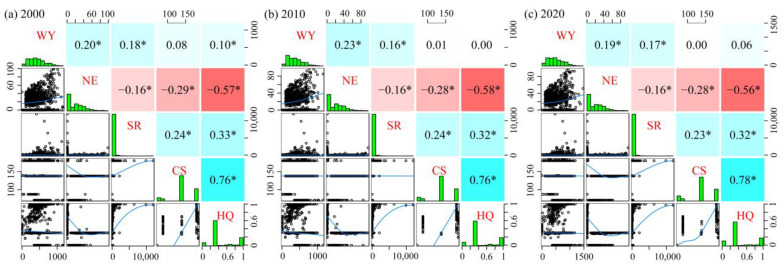
Pearson correlations between different ESs. WY: water yield; NP: nitrogen export; SR: soil retention; CS: carbon storage; HQ: habitat quality; * Correlation significant at *p* < 0.05 (two-tailed).

The relationship and intensity of the five ESs were explored through correlation analysis (Figure 6). Between 2000 and 2020, the synergistic relationship among ESs was dominant, and the trade-off relationship mainly existed between the nitrogen export service and other services. In particular, a strong synergistic effect between carbon storage and habitat quality was observed with a correlation coefficient of as high as 0.76; a strong trade-off between nitrogen exports and habitat quality was observed, where the absolute value of the correlation coefficient was approximately 0.57. Notably, a significant synergistic effect between the water yield and habitat quality existed in 2000 but did not pass the significance test (*p* < 0.05) for other years, indicating spatiotemporal variability in the trade-off and synergistic relationships.

#### 3.3.2. Hotspot Analysis

**Figure 7:**
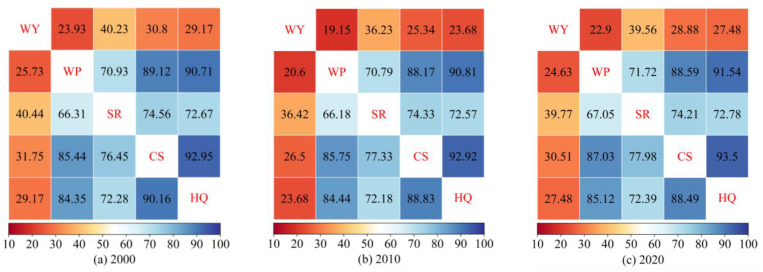
Proportion of overlap of ES hotspots. WP: water purification.

**Figure 8:**
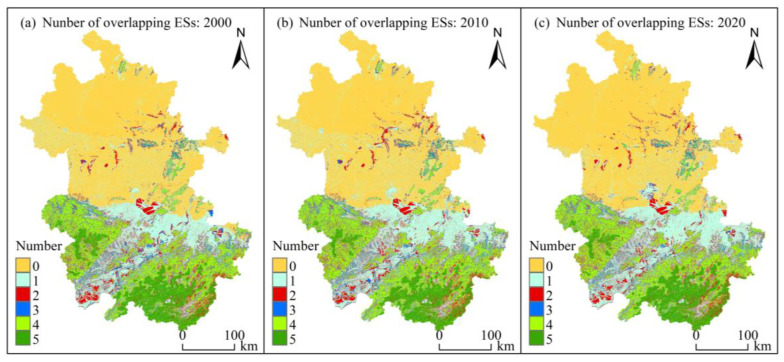
Overlap of different ES hotspots.

During the study period, the spatial overlap percentages of hotspots among different services were similar (Figure 7), and the overlaps of the hotspots among the ESs were relatively high except for the water yield service. Among them, the overlaps of carbon storage with habitat quality and water purification were high, and the overlap between water yield and water purification was the lowest (2010), with the overlapping area only accounting for 19.15% of the water yield hotspots. The spatial overlay distribution of various service hotspots is shown in Figure 8. The ecosystem with the highest capacity to provide comprehensive services per unit area was assigned a value of “5”. The sum of “0” and “1” accounted for 64.75%, 64.67%, and 64.84% of the study area, whereas “5” accounted for only 6.53%, 5.25%, and 6.27% of the study area, with a relatively low percentage of unit areas that could provide multiple ESs at the same time. The clarification of the location of the high-number area has scientific reference value for the identification of priority protected areas and for the adjustment and implementation of ecological projects.

### 3.4. ES simulation and Prediction

**Figure 9:**
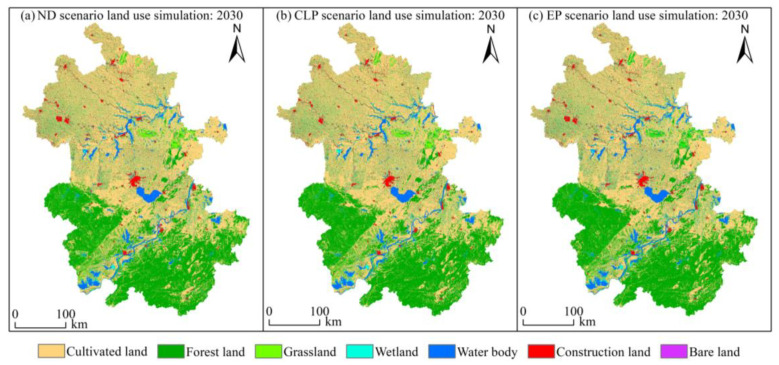
Results from the multi-scenario simulation for land use in 2030.

**Figure 10:**
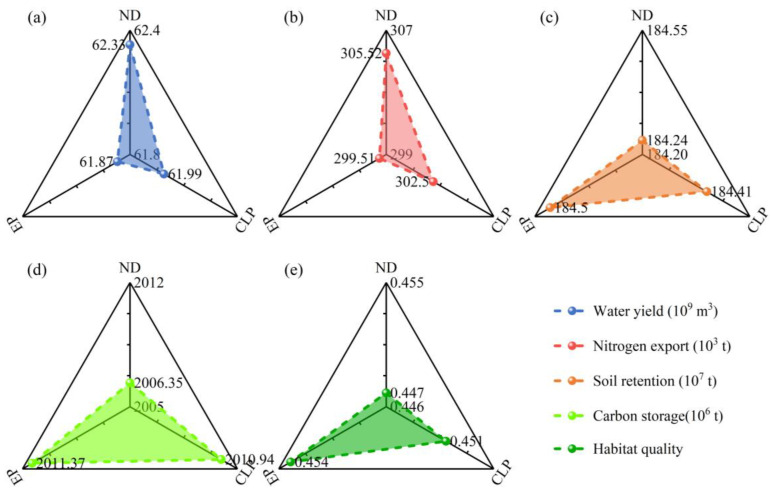
Changes in ESs in 2030 under different scenarios.

Based on the simulation results of land use under different scenarios using the PLUS model and 12 driving factors (Figure 9), the changes in future ESs were analyzed using the InVEST model (Figure 10). The water yield under the ND scenario is higher than under the CLP and EP scenarios but is 4.14 billion m^3^ lower than that in 2020, i.e., a decrease in the future water yield is found. Note that under the ND scenario, the nitrogen export service exhibits a significant increase in the total nitrogen export of 3.34 × 10^3^ t compared to that in 2010 (where the highest value occurs between 2000 and 2020) and the water purification service degrades. In addition, the changes in soil retention, carbon storage, and habitat quality are similar to some extent, all peaking in the development model of the EP scenario, mainly because of the increase in the proportion of forest land, grassland and water body area, the increase in carbon storage and habitat quality and the decrease in total soil erosion due to the limitation of the expansion of construction land.

## 4. Discussion

### 4.1. Analysis of Spatiotemporal ES Changes

ESs are the result of the combined effects of the natural environment and socioeconomic activities [67], and quantifying their spatiotemporal heterogeneity is conducive to exploring the potential of regional development. This study found that the spatial distribution of the same ESs in the past 20 years had some similarity and was correlated with the landscape pattern, indicating that the structure and function of ecosystems were closely related to the spatial allocation of land use [11]. The high-value areas of water yield, soil retention, carbon storage, and habitat quality were mainly located in the Dabieshan and Southern Mountains, and the low-value areas were mainly located in the plains area. The case of the total nitrogen output was the opposite because the mountainous areas are dominated by forest land, a land type that contributes relatively high ESs [68], and has ecological benefits such as controlling soil erosion [69], increasing carbon sequestration [70] and maintaining biodiversity [30], which are beneficial to the sustainable development of the environment. By comparison, plain areas are dominated by cultivated and construction land, which are basic to human survival and development but also pose enormous challenges to realizing the functions and services of the ecosystem [31], such as nutrient export to rivers, which upon exceeding a certain threshold leads to eutrophication and thus a decrease in the richness of aquatic species [71], posing a threat to water ecological security [72]. Therefore, the quantitative analysis of the supply and demand of ESs and their spatial matching relationships can help promote the high-quality development process of regional integration [73], which is the focus of future research.

The PLUS model improves the accuracy of future multi-scenario land simulations and can effectively explore the causes of changes in different land types, which is helpful for the formulation of land pattern optimization policies [32,66]. In this study, three development scenarios, ND, CLP, and EP, were established for use in simulating changes in land use and predicting ESs. Wu et al. [62] evaluated the characteristics of the spatiotemporal evolution of habitat quality in the same province and found that the multiyear average habitat quality was 0.4339. In the present study, the historical and simulated values of the average habitat quality fluctuated in the range of 0.440–0.454. The good agreement between the two indicated the validity and reliability of this study. Additionally, the five ESs found under the CLP scenario in this study were all found to lie between those found under the ND and EP scenarios, i.e., the result was similar to a transition pattern between the ND and EP scenarios. Subsequently, the land use pattern can be further optimized by adjusting the transfer probability of land types to provide a reference for accurate decision-making in the region [74].

### 4.2. Trade-Off Analysis

Coupling different ESs for trade-off analysis helps find a balance between ecological protection and socioeconomics and achieve coordinated regional development. Figure 1c and Figure 5 show that the distribution area of forest land was a low-value area for nitrogen export and a relatively high-value area for other services, indicating that forest land had the function of improving the water quality and enhancing soil and water retention and biodiversity, which is consistent with the analysis of the trade-off relationship among various ESs based on the correlation coefficient method, and a similar pattern has been found for the Songhua River Basin [63]. A significant positive correlation was observed between water yield and nitrogen export, with relatively little overlap between the water yield hotspot and water purification hotspot (Figure 7). This finding was mainly due to the continuous increase in the area of construction land, a land type with a relatively low vegetation cover and a weak evapotranspiration capacity, the increase in the watershed surface runoff, and multiple factors such as the conversion of forest land to cultivated land show a synergistic effect. Hu et al. [75] considered a trade-off relationship between the two, probably due to the different basic units of data statistics, indirectly reflecting the spatiotemporal heterogeneity of the trade-off and synergistic effect among ESs. Unlike traditional “high-value” identification methods (local Moran’s I, local bivariate Moran’s I [28], and Getis-Ord G* statistics [76]), the hotspot analysis method can measure the level of trade-offs between different ESs by analyzing the overlap between high-value areas [77] and can set thresholds based on the financial base and other conditions to effectively establish priority areas, thus realizing multiobjective spatial planning. The steps and computer codes for implementing the method are provided in the Supplementary Material.

### 4.3. Limitations and Uncertainties

By coupling the InVEST model and the PLUS model, the spatiotemporal changes in ESs in the study area and the trade-offs among various services were clarified, and the locations of high-value ES areas were identified, which have reference values for formulating regional land ecological control policies. However, this study still has some limitations and uncertainties. (1) Due to the limitations of human and financial resources, only some parameters of the model were set based on similar regions and the accessibility of results, but no field monitoring statistics were conducted, and the accuracy and timeliness of the parameters must be further improved [37]. (2) A “NoData” value was obtained in the nitrogen export assessment (Figure 5b,g,l) for the water body. The main reason for this finding is that the Nutrient Delivery Ratio model considers the retention and removal capacity of vegetation and soil for nutrient pollutants in the runoff but does not adequately assess the nutrient retention capacity of nonterrestrial water bodies. As a result, although the visualization and evaluation results can be improved by upgrading the resolution of the DEM, the principle of the model must still be improved and optimized. (3) Nature reserves are the areas with the most stringent protection [78], and their designation as spatial policy restriction conversion areas for future land use simulations is scientifically sound. However, due to the influence of resource extraction and development, the streamlining and downgrading of protected areas can lead to changes in the land types of nature reserves [79]. Determining the type of restricted area (e.g., those with ecological protection redline restriction, prohibited development zone restrictions, and permanent basic cultivated area restrictions) that can be more objectively used as a nonpermitted land conversion area to simulate future land types requires further research.

## 5. Conclusions

This study used multisource data to investigate the characteristics of the spatiotemporal evolution of ESs in Anhui Province and the trade-offs among various services. The following conclusions were drawn: (1) During 2000–2020, cultivated land, forest land, and construction land dominated in Anhui Province, with a combined total of more than 92.5% of the study area. Among them, the area of forest land decreased continuously, the proportion of construction land increased, and the habitat was degraded. (2) In the past 20 years, the overall spatial distribution pattern of the ESs was high in the south and low in the north, and the water purification, carbon storage, and habitat quality services declined. (3) The spatial overlay of high-value areas of ESs indicated differences in the capacity of ecosystems per unit of area to provide comprehensive services. The percentage of hotspots with “0” or “1” was more than 64%, suggesting an urgent need to improve the synergistic effect among services to enhance ecological and social benefits. (4) The multi-scenario simulation of land use indicated that in the future, the water yield service will decrease, and the nitrogen output will increase significantly under the natural development scenario, while the other three services will all reach their peaks in the ecological protection scenario. In summary, the ecological protection scenario is more beneficial to improving the overall benefits of ESs.

## Figures and Tables

**Table 1:** Biophysical data used in the InVEST model.

LULC	Cultivated Land	Forest Land	Grassland	Wetland	Water Body	Construction Land	Bare Land
Kc	0.6	1	0.65	0.8	1	0.3	0.5
root_depth	2000	7000	2600	1000	100	500	500
LULC_veg	1	1	1	0	0	0	0
load_n	100	2.8	8	2.8	2.8	100	4
eff_n	0.2	0.8	0.75	0.8	0.8	0.05	0.01
crit_len_n	25	300	100	10	10	10	10
usle_c	0.35	0.003	0.01	0.001	0.001	0.001	0.01
usle_p	0.4	0.2	0.2	0.001	0.001	0.001	0.2
C_above	15.8	44.6	17.7	10.83	8.2	1.2	10.36
C_below	40.3	11.1	44.2	19.18	39.5	27.6	32.4
C_soil	78.2	124.3	124.7	106.7	40.6	43.2	53.8
C_dead	5	1.9	0.08	3.98	0	0	0.96
MAX_DIST	4	-	-	-	-	8	6
WEIGHT	0.6	-	-	-	-	0.4	0.5
DECAY	linear	-	-	-	-	exponential	linear
HABITAT	0.3	1	0.8	0.7	0.7	0	0.6
Cultivated land	0	0.6	0.8	0.55	0.5	0	0.6
Construction land	0.8	0.75	0.6	0.7	0.4	0	0.4
Bare land	0.4	0.2	0.6	0.55	0.2	0.1	0

**Table 2:** Neighbourhood weights.

Land Use Type	Cultivated Land	Forest Land	Grassland	Wetland	Water Body	Construction Land	Bare Land
weight	0.319021357	0.154824223	0.077717415	0.02203639	0.085903028	0.338678453	0.001819133

**Table 3:** Conversion cost matrix.

	ND							CLP							EP						
	a	b	c	d	e	f	g	a	b	c	d	e	f	g	a	b	c	d	e	f	g
a	1	1	1	1	1	1	1	1	0	0	0	0	0	0	1	1	1	1	1	1	1
b	1	1	1	1	1	1	1	1	1	1	1	1	1	1	0	1	1	1	1	0	0
c	1	1	1	1	1	1	1	1	1	1	1	1	1	1	0	1	1	1	1	0	0
d	1	1	1	1	1	1	1	1	1	1	1	1	1	1	0	1	1	1	1	0	0
e	1	1	1	1	1	1	1	1	1	1	1	1	1	1	0	1	1	1	1	0	0
f	1	1	1	1	1	1	1	0	0	0	0	0	1	0	0	0	0	0	0	1	0
g	1	1	1	1	1	1	1	1	1	1	1	1	1	1	1	1	1	1	1	1	1

ND: natural development scenario; CLP: cultivated land protection scenario; EP: ecological protection scenario; a: cultivated land; b: forest land; c: grassland; d: wetland; e: water body; f: construction land; and g: bare land.

**Table 4:** Changes in ESs in Anhui Province from 2000 to 2020.

Type of ES	2000	2010	2020	Changes (2000–2020)
Water yield (10^9^ m^3^)	58.97	50.86	66.47	7.50
Nitrogen export (10^3^ t)	301.02	302.18	299.78	−1.24
Soil retention (10^7^ t)	184.47	184.46	184.57	0.10
Carbon storage (10^6^ t)	2024.46	2012.02	1990.62	−33.84
Habitat quality	0.454	0.453	0.440	−0.014

## Data Availability

Not applicable.

## Supplementary Materials

The following supporting information can be downloaded at: https://www.mdpi.com/article/10.3390/ijerph20010855/s1.
