# Supplementary material for: Spatial-Temporal Variations and Trade-Offs of Ecosystem Services in Anhui Province, China
Source: Int J Environ Res Public Health. 2023 Jan 3;20(1):855. doi: 10.3390/ijerph20010855 (PMC9819523; doi:10.3390/ijerph20010855)
Supplement: Supplementary file 1 [file ijerph-20-00855-s001.zip › Supplementary material.pdf]

Spatial-Temporal Variations and Trade-Offs of Ecosystem Services in Anhui Province, China

Hotspot analysis

Steps and codes to extract high-value areas of ecosystem services (taking carbon storage as an example):

Firstly, the raster map of ecosystem services was obtained with the help of the InVEST model.

Secondly, based on IDLE (Python GUI) to converts a raster dataset to point features. The code is as follows:

```
(import arcpy
from arcpy import env
env.workspace = r"F:\carbon storage\cs2000"
inRaster = r"cs_2000.tif"
outPoint = r"F:\carbon storage result\carbon storage.gdb\cs2000"
field = "VALUE"
arcpy.RasterToPoint_conversion(inRaster, outPoint, field)
print('Point has done!'))
```

Thirdly, output the attribute table of the “cs2000.shp” as a text file, and run the text file with Jupyter notebook, the code is as follows:

```
(import pandas as pd
cf="G:/cs2000/cs2000.txt"
cdf=pd.read_csv(cf)
cdf

cdf = cdf.drop(columns=['OBJECTID','pointid']) #Delete field
cdf

cdf.dropna(subset=['grid_code'],inplace=True) #Delete the row corresponding to the null value
cdf = cdf.drop(cdf[cdf['grid_code']<0].index) #Delete the row corresponding to a negative value
```

```
cdf

cdf=cdf.sort_values(by="grid_code",ascending=False) # Sort the data
cdf

data=cdf[0:x] # take the first x rows of the data to obtain the critical value
data

data.to_csv('G:/cs2000/cs_2000.csv',encoding='utf_8_sig',index=False) # Output running data)
```

Finally, Obtain hotspot raster map with ArcGIS Raster Calculator tool.
